# Matrix Metallopeptidase-Gene Signature Predicts Stage I Lung Adenocarcinoma Survival Outcomes

**DOI:** 10.3390/ijms24032382

**Published:** 2023-01-25

**Authors:** Chia-Hsin Liu, Yuanpu Peter Di

**Affiliations:** 1 Department of Environmental and Occupational Health, School of Public Health, University of Pittsburgh, Pittsburgh, PA 15261, USA; 2 Division of Pulmonary and Critical Care Medicine, Department of Internal Medicine, Tri-Service General Hospital, National Defense Medical Center, Taipei 11490, Taiwan

**Keywords:** matrix metallopeptidase, lung adenocarcinoma, gene signature, *KRAS*, *EGFR*

## Abstract

Tumor recurrence poses a significant challenge to the clinical management of stage I lung adenocarcinoma after curative surgical resection. Matrix metalloproteinases (MMPs) increase expression and correlate with recurrence and metastasis in surgically resected non-small cell lung cancer. However, the impact of MMPs on survival outcome varies, and their roles in patients with stage I lung adenocarcinoma remain unclear. In two discovery cohorts, we first analyzed 226 stage I–II lung adenocarcinoma cases in the GSE31210 cohort using a clustering-based method and identified a 150-gene MMP cluster with increased expression in tumors associated with worse survival outcomes. A similar analysis was performed on 517 lung adenocarcinoma cases in the Cancer Genome Atlas cohort. A 185-gene MMP cluster was identified, which also showed increased expression in tumors and correlated with poor survival outcomes. We further streamlined from the discovery cohorts a 36-gene MMP signature significantly associated with recurrence and worse overall survival in patients with stage I lung adenocarcinoma after surgical resection. After adjusting for covariates, the high MMP-gene signature expression remained an independent risk factor. In addition, the MMP-gene signature showed enrichment in epidermal growth factor receptor wild-type lung tumors, especially for those with Kirsten rat sarcoma virus mutations. Using an independent validation cohort, we further validated the MMP-gene signature in 70 stage I lung adenocarcinoma cases. In conclusion, MMP-gene signature is a potential predictive and prognostic biomarker to stratify patients with stage I lung adenocarcinoma into subgroups based on their risk of recurrence for aiding physicians in deciding the personalized adjuvant therapeutics.

## 1. Introduction

Lung cancer is the leading cancer-related death worldwide, with a 5-year survival rate of 22% [1]. About 80% of lung cancer patients are diagnosed with locally advanced or metastatic disease when curative surgery is no longer feasible [2]. Regardless of curative surgery for early-stage lung cancer, 20% to 40% of stage I patients will have tumor recurrence, which remains the leading cause of cancer-related death [3,4,5,6,7,8]. Patients with stage I lung adenocarcinoma, the most common histological subtype, vary in survival outcomes [8,9]. This indicates that the current TNM staging system fails to distinguish patients at a higher risk of recurrence for stage I lung cancer following surgical resection [10]. 

Although adjuvant chemotherapy has been shown to decrease tumor recurrence and prolong survival in completely resected stage II or III non-small cell lung cancer (NSCLC), its role in stage I disease remains controversial [11]. The current National Comprehensive Cancer Network guidelines for NSCLC recommend postoperative chemotherapy for patients with stage IB (T2a, N0) disease and negative surgical margins who have high-risk features, including tumors larger than 4 cm, poorly differentiated tumors, vascular invasion, visceral pleural involvement, wedge resection, and unknown lymph node status (Nx) [12]. Furthermore, Osimertinib is also recommended as an adjuvant therapy option for eligible patients with completely resected stage IB to IIIA NSCLC with epidermal growth factor receptor (*EGFR*) exon 19 deletions or L858R mutations who have previously received adjuvant chemotherapy or are ineligible to receive platinum-based chemotherapy [13]. Nevertheless, previous studies showed that patients with stage I disease did not benefit from adjuvant chemotherapy after surgical resection except for survival advantage for stage IB patients who had tumor size ≥4 cm [14]. This is probably because patients with stage I disease and a low risk of recurrence may not benefit from routine adjuvant chemotherapy. However, patients with high-risk factors such as large tumor size (≥4 cm) demonstrated a significant survival difference in favor of adjuvant chemotherapy. This highlights the need for reliable predictive biomarkers to stratify high-risk stage I disease for adjuvant chemotherapy.

Degradation of the extracellular matrix and penetration of the basement membrane have been shown to involve in tumor invasion and metastasis [15,16]. The matrix metalloproteinases (MMPs) are a family of 24 proteolytic enzymes that degrade the extracellular matrix and are involved in many phases of cancer progression, including invasiveness, angiogenesis, and metastasis [16,17,18,19]. Studies have shown that high levels of MMPs were expressed in lung tumors and correlated with tumor recurrence and poor survival outcomes in patients with surgically resected non-small cell lung cancer (NSCLC) [20,21,22,23,24,25,26,27,28]. In addition, each MMP can degrade multiple substrates, and many substrates are degraded by various MMPs, suggesting that multiple MMPs may involve in either physiological processes or disease progression such as cancer [29]. However, previous studies assessed the individual MMP as a prognostic marker, which may overlook the effect of co-expressed MMPs and related genes on survival outcomes, leading to inconsistent results [30]. Moreover, the function of different types of MMPs vary, and their effects on survival outcomes for stage I lung adenocarcinoma remain unknown.

In this study, we analyzed two publicly available lung adenocarcinoma datasets from GSE31210 and the Cancer Genome Atlas using the clustering-based method and discovered MMP-enriched gene clusters. A streamlined 36-gene molecular signature was identified from these two MMP-gene clusters as a potential biomarker to predict survival outcomes in patients with stage I lung adenocarcinoma after complete resection. Finally, we validated the utility of the predictive and prognostic transcriptome MMP-gene signature in an independent cohort.

## 2. Results

### 2.1. MMPs Overexpress in Lung Tumors and Correlate with Survival Outcomes in the GSE31210 Cohort

**Figure 1:**
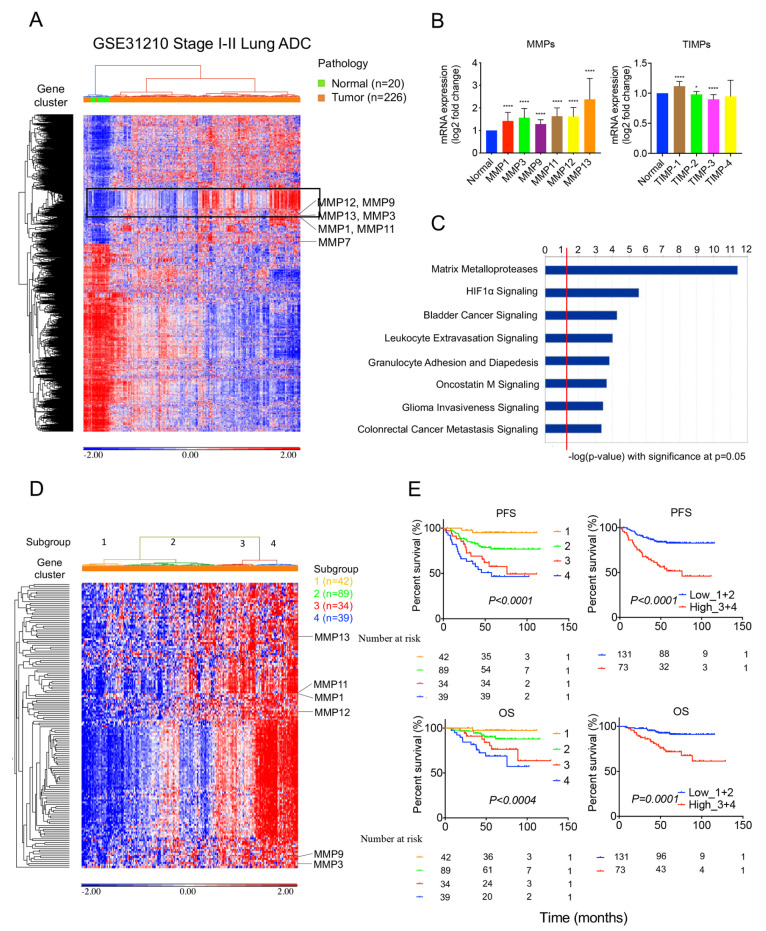
MMPs increase expression in lung tumors and are associated with survival outcomes in the GSE31210 cohort; 226 lung adenocarcinoma patient samples and 20 normal lung samples from the GSE31210 stage I–II lung adenocarcinoma cohort were analyzed: (**A**) An unsupervised hierarchical clustering heatmap of differentially expressed genes between tumor and normal lung tissue was performed. A 150-gene cluster with enriched MMPs was identified (square). (**B**) The MMPs, including *MMP1*, *MMP3*, *MMP7*, *MMP9*, *MMP11*, *MMP12*, and *MMP13*, increased expression in tumors compared to normal lung tissue. *TIMP-1* increased expression in tumors, but *TIMP-2* and *TIMP-3* decreased expression in tumors compared to normal lung tissue. Data shown are mean ± S.D. **** *p* < 0.0001 and * *p* < 0.005 using Student’s *t* test. (**C**) Ingenuity pathway analysis of the 150-gene cluster showed significant pathways. (**D**) Unsupervised hierarchical clustering heatmap revealed four lung adenocarcinoma subgroups clustered with 150-gene cluster (**E**) Kaplan–Meier survival analysis of patient subgroups based on the 150-gene cluster was performed using the log-rank test.

To examine MMP expression in lung tumors, we analyzed the gene expression of 226 lung adenocarcinoma samples and 20 normal lung samples from the GSE31210 stage I–II lung adenocarcinoma cohort (Supplementary Table S1). We performed an unsupervised hierarchical clustering analysis showing the heatmap of differentially expressed genes (absolute fold change >2.5, FDR < 0.05) between tumor and normal tissue (Figure 1A) in this cohort. The results showed a 150-gene cluster with enriched MMPs, including *MMP1, MMP3, MMP9, MMP11, MMP12,* and *MMP13* (Figure 1A and Supplementary Table S2) and increased expression in tumors compared to normal lung tissue (Figure 1B). In addition, tissue inhibitors of metalloproteinases (TIMPs) such as *TIMP-1* increased expression in tumors, but *TIMP-2* and *TIMP-3* decreased expression in tumors in contrast to normal lung tissue (Figure 1B). The ingenuity pathway analysis of the 150-gene cluster showed significant pathways associated with the participation of MMPs (Figure 1C and Supplementary Table S3). An unsupervised hierarchical clustering heatmap revealed four lung adenocarcinoma subgroups clustered with the 150-gene cluster (Figure 1D). Overall, the MMP-gene cluster expression from subgroup 1 to subgroup 4 was consistent with the expression pattern from low to high. In addition, patients with higher gene cluster expression correlated with worse progression-free survival (PFS) and overall survival (OS) than those with lower expression (Figure 1E and Supplementary Figure S1A).

### 2.2. MMPs Overexpress in Lung Tumors and Correlate with Survival Outcomes in TCGA Cohort

**Figure 2:**
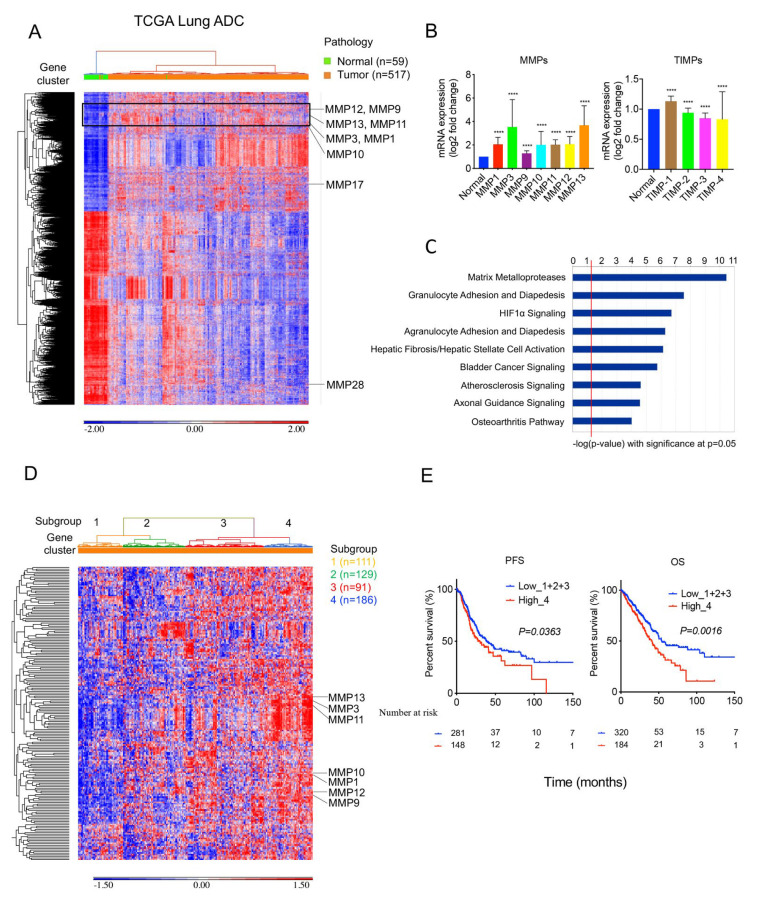
MMPs increase expression in lung tumors and are associated with survival outcomes in the TCGA cohort; 517 lung adenocarcinoma patient samples and 59 normal adjacent lung samples from the TCGA lung adenocarcinoma cohort were analyzed: (**A**) An unsupervised hierarchical clustering heatmap of differentially expressed genes between tumor and normal lung tissue was performed. A 185-gene cluster with enriched MMPs was identified (square). (**B**) The MMPs, including *MMP1*, *MMP3*, *MMP9*, *MMP11*, *MMP12*, and *MMP13*, increased expression in tumors more than normal lung tissue. *TIMP-1* increased expression in tumors, but *TIMP-2*, *TIM-3*, and *TIMP-4* decreased expression in tumors compared with normal lung tissue. Data shown are mean ± S.D. **** *p* < 0.0001 using Student’s *t* test. (**C**) Ingenuity pathway analysis of the 185-gene cluster showed significant pathways. (**D**) An unsupervised hierarchical clustering heatmap revealed four lung adenocarcinoma subgroups clustered with a 185-gene cluster. (**E**) Kaplan–Meier survival analysis of patient subgroups based on a 185-gene cluster was performed using the log-rank test.

To validate the results from the GSE31210 cohort, we performed a transcriptome analysis of the Cancer Genome Atlas (TCGA) lung adenocarcinoma cohort (Supplementary Table S4), which included 517 lung tumor samples and 59 adjacent normal lung tissue samples. The unsupervised hierarchical clustering heatmap based on the differentially expressed genes (absolute fold change >2.5, FDR < 0.05) between the tumor and the adjacent normal lung tissue (Figure 2A). Consistently, the result showed a 185-gene cluster with enriched MMPs, including *MMP1, MMP3, MMP9, MMP10, MMP11, MMP12,* and *MMP13* (Figure 2A and Supplementary Table S2), showing increased expression in tumors than adjacent normal lung tissue (Figure 2B). Furthermore, *TIMP-1* increased expression in tumors, but *TIMP-2*, *TIM-3*, and *TIMP-4* decreased expression in tumors compared with normal lung tissue (Figure 1B). Similar to the results from the GSE31210 cohort, ingenuity pathway analysis of the 185-gene cluster revealed significant pathways mainly involving MMPs in the TCGA lung adenocarcinoma cohort (Figure 2C and Supplementary Table S5). An unsupervised hierarchical clustering heatmap revealed four lung adenocarcinoma subgroups clustered with the 185-gene cluster (Figure 2D). Similarly, patients with higher MMP-gene cluster expression correlated with worse PFS and OS than those with lower expression (Figure 2E and Supplementary Figure S2A).

### 2.3. Development of a 36-Gene MMP Signature and Network Analysis

**Figure 3:**
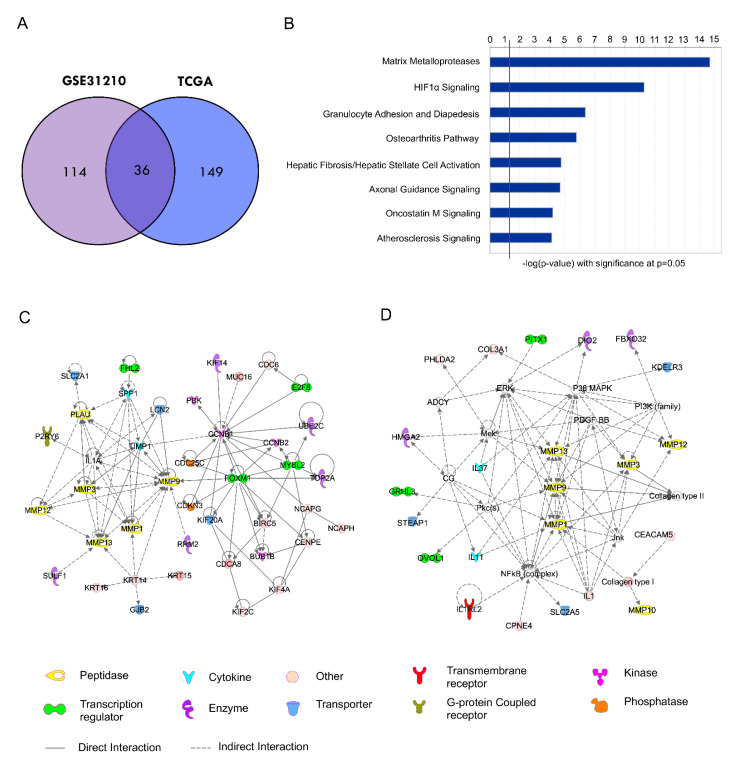
Characteristics of a 36-gene MMP signature and network analysis: (**A**) Venn diagram showed 36 MMP-associated genes, which were shared between MMP-enriched gene clusters in GSE31210 and TCGA cohort. (**B**) Ingenuity pathway analysis of the 36-gene MMP signature showed significant pathways. (**C**) Networks analysis of the 150-gene cluster from the GSE31210 cohort. (**D**) Networks analysis of the 185-gene cluster from the TCGA cohort.

To search for more stable molecular signatures associated with MMPs in these two cohorts, we identified 36 overlapping genes from the MMP-related gene clusters between GSE31210 and TCGA cohort, termed the 36-gene MMP signature (Figure 3A and Supplementary Table S2). Ingenuity pathway analysis of the 36-gene MMP signature also showed significant pathways with enrichment related to MMPs (Figure 3B and Supplementary Table S6). The network analysis of the 150-gene cluster from the GSE31210 cohort showed the prediction of critical upstream regulators such as MYBL2, E2F8, FOXM1, and FHL2 to regulate this interaction network transcriptionally (Figure 3C). In addition, the 185-gene cluster from the TCGA cohort revealed the network’s connection of MAPK/ERK and NFκB pathway (Figure 3D).

### 2.4. High 36-Gene MMP Signature Expression Predicts Poor Survival Outcomes in GSE3120 Stage I Lung Adenocarcinomas

**Figure 4:**
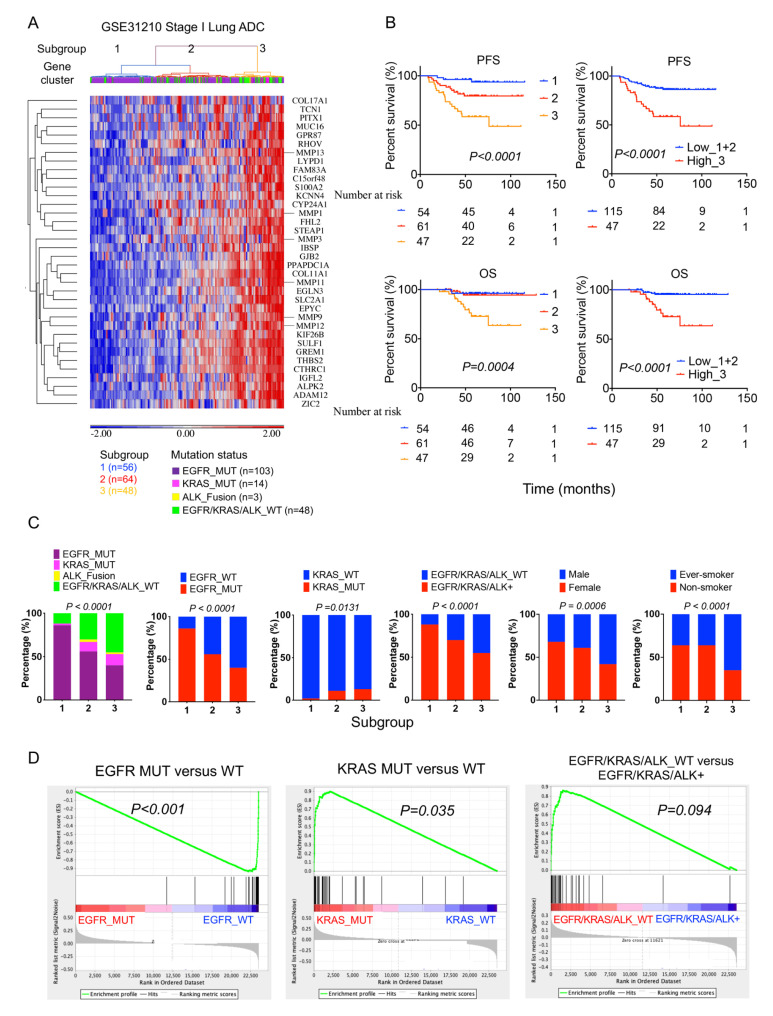
High MMP gene signature expression predicts poor survival outcomes in stage I lung adenocarcinoma from the GSE31210 cohort; 168 stage I lung adenocarcinoma patient samples from the GSE31210 cohort were analyzed: (**A**) An unsupervised hierarchical clustering heatmap revealed three lung adenocarcinoma subgroups clustered with the 36-gene MMP signature. (**B**) Kaplan–Meier survival analysis of patient subgroups based on the 36-gene MMP signature was performed using the log-rank test. (**C**) The distribution difference among the three subgroups stratified by gene mutation, gender, and smoking status was tested by the Chi-squared test. (**D**) GSEA showed the enrichment of 36-gene MMP signature based on mutation status in the GSE31210 cohort.

To determine the prognostic value of the streamlined 36-gene MMP signature in stage I lung adenocarcinoma cases, we performed unsupervised hierarchical clustering heatmap analysis of the gene signature and stage I lung adenocarcinoma from the GSE31210 cohort. The result revealed three lung adenocarcinoma subgroups clustered with the gene signature (Figure 4A). In survival analysis, patients with higher gene signature expression were associated with worse PFS and OS than those with lower expression (Figure 4B and Supplementary Figure S1B). Notably, the patient cluster with increased MMP gene signature expression remained an independent risk factor for PFS and OS in patients with stage I lung adenocarcinoma after adjustment of other covariates, including stage, gender, age, smoking, and mutation status (Supplementary Table S7). We also analyzed the gene mutation, gender, and smoking status in these subgroups, and the results revealed that lung cancer subgroups with higher MMPs-gene signature expression were associated with a higher proportion of *EGFR* wild type, Kirsten rat sarcoma virus (*KRAS*) mutation, triple-negative mutations, male, and ever-smoker (Figure 4C). Consistently, the GSEA showed that the 36-gene MMP signature enriched in *EGFR* wild-type lung tumors, especially in *KRAS*-driven lung tumors (Figure 4D).

### 2.5. High 36-Gene MMP Signature Expression Predicts Poor Survival Outcomes in TCGA Stage I Lung Adenocarcinomas

**Figure 5:**
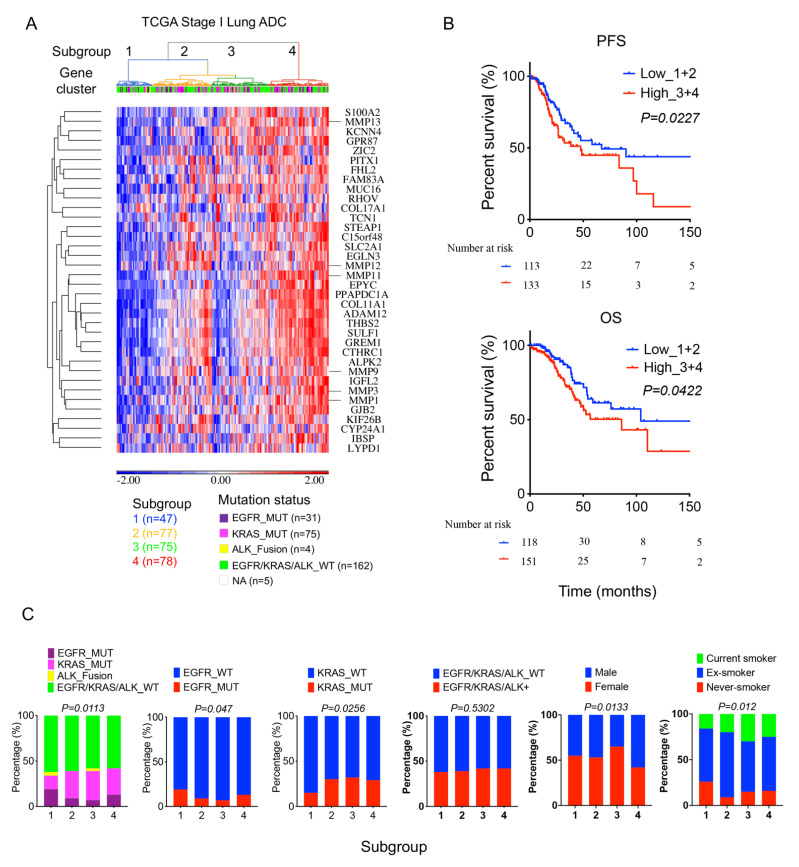
High MMP gene signature expression predicts poor survival outcomes in stage I lung adenocarcinoma from the TCGA cohort; 277 stage I lung adenocarcinoma patient samples from the TCGA cohort were analyzed: (**A**) An unsupervised hierarchical clustering heatmap revealed four lung adenocarcinoma subgroups clustered with 36-gene MMP signature. (**B**) Kaplan–Meier survival analysis of patient subgroups based on the 36-gene MMP signature performed using the log-rank test. (**C**) The distribution difference among the four subgroups stratified by gene mutation, gender, and smoking status was tested by the Chi-squared test.

We further used the 36-gene MMPs signature to analyze the TCGA stage I lung adenocarcinoma cases. Unsupervised hierarchical clustering heatmap of the gene signature and the stage I cases showed four lung adenocarcinoma subgroups (Figure 5A). In survival analysis, patients with higher MMPs-gene signature expression correlated with poor survival outcomes than those with lower expression (Figure 5B and Supplementary Figure S2B). We also analyzed the gene mutation, gender, and smoking status in these subgroups. The results revealed that mutation statuses were differentially distributed between subgroups, suggesting that the MMP-gene signature was associated with driver oncogenes. (Figure 5C). Furthermore, a higher proportion of males and smoking history were correlated with higher MMP-gene signature expression in lung cancer subgroups (Figure 5C).

### 2.6. A 36-Gene MMPs Signature Is Validated in An Independent Lung Cancer Cohort

**Figure 6:**
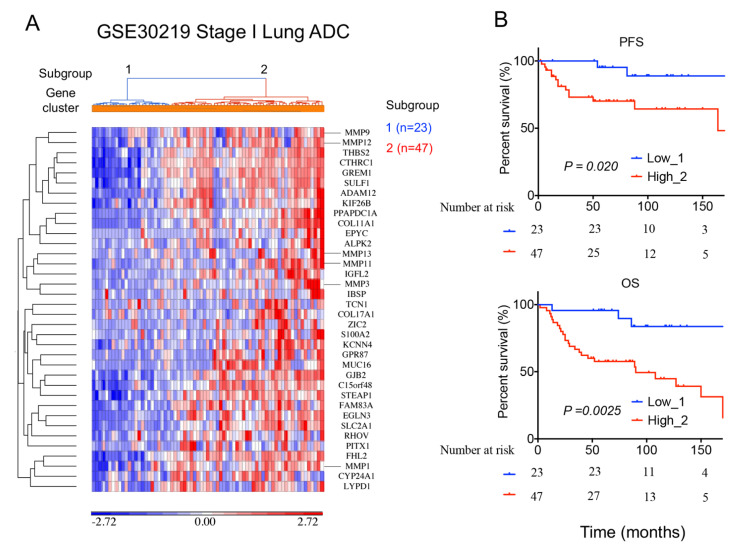
Validation of the 36-gene MMP signature in an independent GSE30219 lung cancer cohort; 70 stage I lung adenocarcinoma patient samples from the GSE30219 cohort were analyzed: (**A**) An unsupervised hierarchical clustering heatmap revealed two lung adenocarcinoma subgroups clustered with 36-gene MMP signature. (**B**) Kaplan–Meier survival analysis of patient subgroups based on the 36-gene MMP signature was performed using the log-rank test.

To validate the 36-gene MMPs signature in an independent lung cancer cohort, we analyzed 70 stage I (T1N0M0) lung adenocarcinoma cases from the GSE30219 cohort (Supplementary Table S8). The unsupervised hierarchical clustering heatmap of the gene signature and stage I cases showed two lung adenocarcinoma subgroups (Figure 6A). Consistently, patients with higher MMP-gene signature expression correlated with worse PFS and OS compared to those with lower expression (Figure 6B).

## 3. Discussion

Surgical resection is the primary curative therapeutic option in patients with stage I lung adenocarcinoma. However, tumor recurrence remains one of the leading causes of cancer-related deaths, highlighting the urgent need for accurate predictive biomarkers to improve clinical management of stage I lung adenocarcinoma after surgical resection. In this study, we performed transcriptome analysis using a clustering-based method. We discovered MMP-enriched gene clusters, which increased expression in tumors compared to normal lung tissue and correlated with poor prognosis in two independent lung adenocarcinoma cohorts. We further identified a 36-gene MMP-gene signature from these two MMP-gene clusters, which showed enrichment in *EGFR* wild-type lung tumors, especially for those with *KRAS* mutations. In addition, the high MMP-gene signature expression independently predicts recurrence and poor overall survival in patients with stage I lung adenocarcinoma after complete resection. Finally, an independent cohort was used to validate the MMP-gene signature’s robustness successfully.

Although individual MMPs have been reported to correlate with survival outcomes in patients with surgically resected NSCLC, these results were inconsistent or not reproducible in different cohorts [31,32,33,34,35,36,37,38,39,40,41], and their effects on stage I lung adenocarcinoma remain unclear. We hypothesized that various MMPs were heterogeneously expressed in lung adenocarcinoma, and their effects on survival outcomes varied. Here, we performed unsupervised hierarchical clustering of DEGs between tumor and normal lung tissue in two lung adenocarcinoma cohorts. Our results showed MMP-enriched gene clusters containing *MMP1, MMP3, MMP9, MMP11, MMP12,* and *MMP13* in these two cohorts. Notably, these MMPs were clustered together and overexpressed in lung tumors compared to normal lung tissue, suggesting that they display similar gene expression patterns and may be functionally related during lung tumorigenesis. We also analyzed tissue inhibitors of metalloproteinases (*TIMPs*) expression, including four paralogues (*TIMP1–TIMP4*) initially characterized as inhibitors of MMPs [42]. *TIMP-1* increased expression in tumors compared to normal lung tissue in the cohorts. In contrast, *TIMP-2*, *TIMP-3*, and *TIMP-4* showed lower expression in the tumors. These results demonstrated that *TIMP* expression, like *MMP* expression, varied in lung adenocarcinoma and has been reported to correlate with survival outcomes [43,44,45].

Consistent with this assumption, the network analysis also demonstrated direct and indirect interaction among MMPs and associated genes within the gene clusters. Therefore, using individual MMP as a predictive or prognostic biomarker to predict survival outcomes may not be sufficient or representative, contributing to inconsistent results in the current knowledge. Furthermore, adjuvant chemotherapy and radiotherapy also influence survival outcomes and may affect survival analysis in surgically resected NSCLC. Therefore, we performed the survival analysis of patients with stage I lung adenocarcinoma who received surgical resection without adjuvant chemotherapy or radiotherapy in the GSE31210 and GSE30219 cohorts. Despite the incomplete data of adjuvant therapy after surgery in the TCGA cohort, the result was consistent with other cohorts in that the higher MMP-gene signature expression was associated with recurrence and poor overall survival in patients with surgically resected stage I lung adenocarcinoma.

Our results showed that the 36-gene MMP signature displayed enrichment in *EGFR* wild-type lung tumors, especially for those with *KRAS* mutations compared to *ALK* translocation and triple-negative mutations, suggesting that these signature genes may be associated with *KRAS*-driven pathways. Consistently, lung cancer subgroups with higher MMP-gene signature expression in the cohorts have a higher proportion of male and smoking history, which has been linked to *KRAS* mutations [46,47]. Previous studies have shown that patients with stage I lung adenocarcinoma and *KRAS* mutations have a significantly higher risk of recurrence than those without the mutation [48,49]. In agreement, a recent meta-analysis suggested that *KRAS* mutations are associated with poor survival outcomes, especially in patients with lung adenocarcinoma and stage I disease [50]. These findings may be partly explained by the high MMP-gene signature expression associated with lung adenocarcinoma with *KRAS* mutations and correlated with recurrence and worse overall survival, as seen in our results. Remarkably, the patients in the GSE31210 cohorts showed a higher *EGFR* mutation rate (61%) compared to the TCGA cohort (11%), which is consistent with the Asians with higher *EGFR* mutation rates in lung adenocarcinoma such as the GSE31210 cohort conducted in Japan [51]. Nonetheless, the MMP-gene signature remained robust to predict survival outcomes regardless of the heterogenicity, such as ethnicity, mutation status, and different platforms within these cohorts.

Several MMPs inhibitors (MMPIs) were developed and used to treat various cancer types in clinical trials during the late 1990s and early 2000s [52,53,54]. Even though the MMPIs showed promising effects in blocking tumor growth and metastasis in preclinical studies, clinical trials of these drugs were not successful [55,56,57]. Multiple reasons have been postulated for the explanation, including the difference between human and murine biology, the non-specificity of MMPIs, and the drug administration being at an advanced stage [58]. Preclinical testing reflected that MMPIs successfully inhibited early-stage cancers and hematogenous metastases while having less effect on large tumors [56]. It has been proposed that new trials should be designed to use MMPIs in patients with early-stage cancers and a high risk of metastasis after surgery or as neoadjuvant therapy prior to surgery [58]. The MMP-gene signature may be a useful predictive biomarker for future clinical trials to identify patients with early-stage lung adenocarcinoma and a high risk of recurrence for MMPI treatment after curative surgery.

## 4. Materials and Methods

### 4.1. Patient and Expression Data

#### 4.1.1. GSE31210 Cohort

The microarray expression data and clinical data were previously obtained under an IRB-approved protocol with informed consent, and downloaded from the National Center for Biotechnology Information Gene Expression Omnibus database (http://www.ncbi.nlm.nih.gov/geo, accessed on 18 April 2019) [59]. Raw gene-expression data were normalized by MAS5. A total of 226 lung adenocarcinoma cases comprised 168 stage I and 58 stage II cases, and 20 normal lung tissue were subjected to expression profiling (Supplementary Table S1). The 204 cases who received complete resection with free resection margins and no involvement of mediastinal lymph nodes and did not receive postoperative chemotherapy and/or radiotherapy, unless relapsed, were subjected to survival analyses. Twenty-two cases were excluded from prognosis analysis due to incomplete resection or adjuvant therapy.

#### 4.1.2. TCGA Cohort

TCGA lung adenocarcinoma RNAseq and clinical data were previously obtained under an IRB-approved protocol with informed consent, and downloaded from UCSC Xena (http://xena.ucsc.edu/, accessed on 4 May 2019) [60]. A total of 517 lung adenocarcinoma cases and 59 adjacent lung tissue were subjected to expression profiling (Supplementary Table S4). Among the 517 cases, 277 stage I cases with survival data were subjected to survival analysis.

#### 4.1.3. GSE30219 Cohort

The microarray expression data and clinical data were previously obtained under an IRB-approved protocol, with informed consent, and downloaded from the National Center for Biotechnology Information Gene Expression Omnibus database (http://www.ncbi.nlm.nih.gov/geo, accessed on 5 May 2019) [61]. Raw gene-expression data were normalized by robust multi-array average (RMA). A total of 70 stage I (T1N0M0) lung adenocarcinoma cases who received surgery and did not receive postoperative chemotherapy and/or radiotherapy selected from 293 lung cancer cases were subjected to survival analysis (Supplementary Table S8).

### 4.2. Ingenuity Pathway Analysis (IPA)

MMP-related gene clusters and signatures, including 150-gene cluster, 185-gene cluster, and 36-gene MMP signature, were used to carry out gene set enrichment analysis using Ingenuity pathway analysis (IPA, http://www.ingenuity.com, accessed on 8 May 2019). IPA was used to determine which pathways were differentially represented in the identified significant genes, compared to the Ingenuity knowledge base.

### 4.3. Gene Set Enrichment Analysis (GSEA)

GSEA was applied using ranked lists of genes from the GSE31210 cohort based on mutation status and sorted by Signal2Noise. After Kolmogorov–Smirnoff testing, a 36-gene MMP signature showing a *P* < 0.05 was considered enriched between mutation status under comparison.

### 4.4. Bioinformatic and Statistical Analysis

Differential expression analysis between tumor and normal lung tissue was performed using Partek Genomics Suite (Partek, St. Louis, MO, USA), and the Benjamini–Hochberg method was used to adjust the raw *P* values for multiple testing. Only genes with fold change (up- and down-regulated) >2.5 and FDR < 0.05 were considered as differentially expressed genes (DEGs). *MMP* and *TIMP* expression between tumor and normal lung tissue were compared by Student’s *t* test. A hierarchical clustering heatmap was conducted using Partek Genomics Suite. Survival was compared using Kaplan–Meyer analysis. The stratification of signature as high or low depends on the expression level with significant differences in the survival outcomes and the lowest log-rank *P* value among subgroups. The log-rank test was used to compare survival or event-free survival between groups, and Cox proportional hazards modeling was used for univariate and multivariate analyses. Chi-squared test was used to compare frequencies in one or more categories. *P* < 0.05 was considered significant.

## 5. Conclusions

In summary, we analyzed transcriptome data of lung adenocarcinoma cases in two discovery cohorts using a clustering-based approach and discovered MMP-enriched gene clusters. A streamlined 36-gene MMPs signature was further identified and successfully predicted recurrence and worse overall survival in patients with stage I lung adenocarcinoma after curative surgery in discovery and validation cohorts. These results will be necessary for the proper stratification of early-stage patients with a high risk of disease recurrence and worse overall survival for optimized follow-up schedules and the use of adjuvant therapeutics such as chemotherapy or MMPIs in future clinical trials.

## Data Availability

The datasets generated and/or analyzed are available as Supplementary Materials in the current study.

## Supplementary Materials

The following supporting information can be downloaded at: https://www.mdpi.com/article/10.3390/ijms24032382/s1.
