# Supplementary material for: Matrix Metallopeptidase-Gene Signature Predicts Stage I Lung Adenocarcinoma Survival Outcomes
Source: Int J Mol Sci. 2023 Jan 25;24(3):2382. doi: 10.3390/ijms24032382 (PMC9917043; doi:10.3390/ijms24032382)
Supplement: Supplementary file 1 [file ijms-24-02382-s001.zip › ijms-2143257-supplementary.pdf]

Matrix Metallopeptidases-Gene Signature Predicts Stage I Lung Adenocarcinoma Survival Outcomes

Chia-Hsin Liu and Yuanpu Peter Di

ONLINE DATA SUPPLEMENT

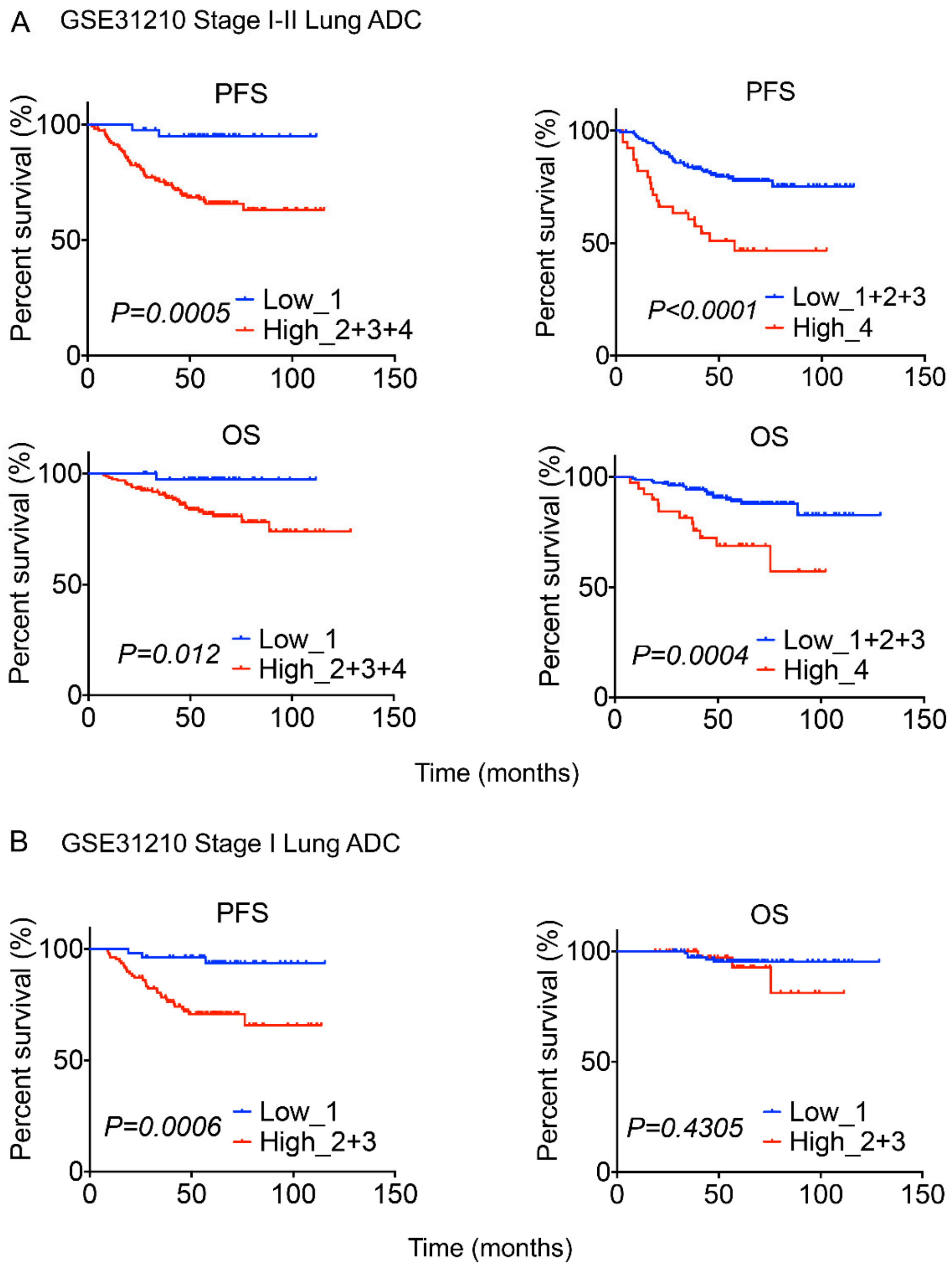

Figure S1. MMPs-gene cluster and 36-gene MMP signature predict survival outcome in GSE31210 cohort. Subgroups of patients with lung adenocarcinoma were determined by unsupervised hierarchical clustering. **(A)** Kaplan–Meier survival analysis based on MMPs-gene cluster in different subgroups. **(B)** Kaplan–Meier survival analysis based on 36-gene MMP signature in different subgroups. *P* value was based on the log-rank test.

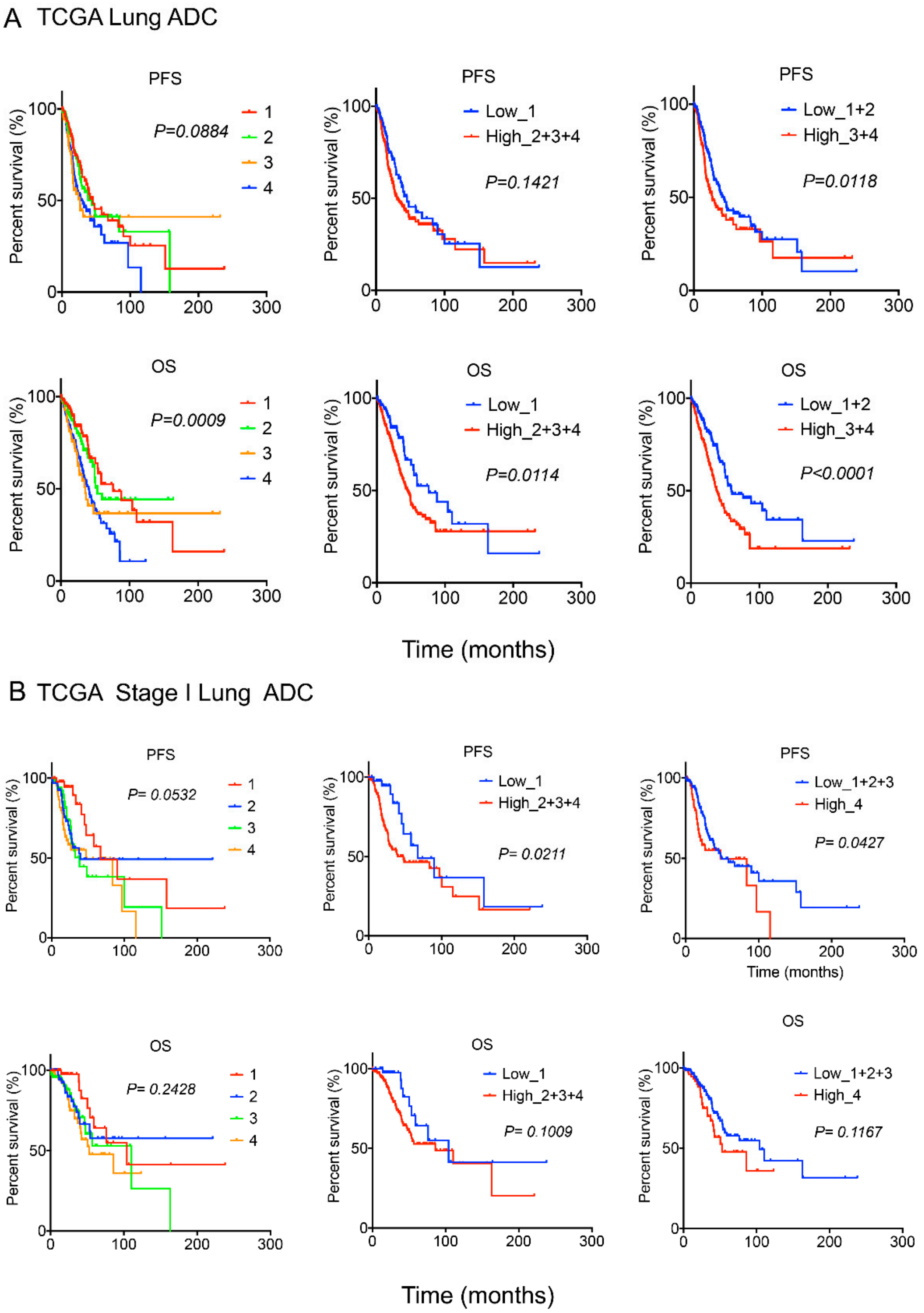

Figure S2. MMPs-gene cluster and 36-gene MMP signature predict survival outcome in TCGA cohort. Subgroups of patients with lung adenocarcinoma were determined by unsupervised hierarchical clustering. **(A)** Kaplan–Meier survival analysis based on MMPs-gene cluster in different subgroups. **(B)** Kaplan–Meier survival analysis based on 36-gene MMP signature in different subgroups. P value was based on the log-rank test.

Table S1. Clinical Characteristics of GSE31210 Stage I-II Lung Adenocarcinoma Cohort .

**Characteristics**	***N* (%)**
Subjects, *n*	226
Sex, M/F, *n* (%)	105/121 (46/54)
Age, yr, mean ± SD	60 ± 7
pTNM stages*	
IA	114 (50)
IB	54 (24)
II	58 (26)
Smoking status	
Non-smoker	115 (51)
Ever-smoker	111 (49)
Mutation status	
EGFR_MUT	127 (56)
KRAS_MUT	20 (9)
ALK _Fusion	11 (5)
KRAS/EGFR/AKL_WT	68 (30)
Exclude for prognosis analysis	22 (9.7)

Clinical–pathologic characteristics of GSE31210 lung adenocarcinoma cohort (n=226) were evaluated in this study. 22 cases were excluded for prognosis analysis due to incomplete resection or adjuvant therapy. *According to the 7th edition of the TNM classification for lung cancer. *Definition of abbreviations:* ALK= Anaplastic lymphoma kinase; EGFR= epidermal growth factor receptor; MUT = mutation; pTNM stages = pathologic TNM stages of malignant tumors; WT = wild type.

Table S2. Gene List of MMP related Gene Cluster and Signature.

Name	Genes
GSE31210 MMPs-gene cluster (n=150)	COL11A1, MMP13, MMP12, GJB2, CYP24A1, GREM1, MMP11, MMP1, IGFL2, MMP3, MUC16, MMP9, CTHRC1, COL17A1, KIF26B, GPR87, ZIC2, IBSP, KCNN4, THBS2, C15orf48, FAM83A, S100A2, SULF1, EPYC, PPAPDC1A, ALPK2, PITX1, SLC2A1, ADAM12, STEAP1, EGLN3, RHOV, FHL2, LYPD1, TCN1, SPP1, KRT15, KRT6A, CDCA8, TOP2A, UBE2T, EXO1, CDC45, E2F8, RAD54L, AKR1B10, ANLN, ARL14, HMMR, MUC5B, CLDN10-AS1, DLGAP5, MB, PTPRH, DEPDC1B, RRM2, ERCC6L, MELK, MYBL2, P2RY6, CLDN1, LRRC15, DEPDC1, IGF2BP3, KIAA0101, S100P, LCN2, KRT6A, TPX2, SCG5, BUB1B, LY6D, CENPA, CENPM, CEP55, ORC1, KIF4A, IL4I1, SLCO5A1, NEIL3, PSAT1, BPIFA1, MND1, NUF2, LINC00525, CHST6, ASPM, ORC6, ZG16B, FOXM1, KIF2C, FAM111B, GINS1, BIRC5, NCAPH, TRAIP, STIL, CYP26A1, KRT16, KIFC1, TTK, SEMA4B, HJURP, SYNGR3, ALDH3B2, ZWINT, CCNB1, APELA, KIF20A, CENPF, NCAPG, KRT14, KRT17, KIF14, SPAG4, LINC00857, NUSAP1, NEK2, PKIB, FAM101A, GPR84, CKAP2L, UHRF1, CFB, CKMT1B, PLAU, BRIP1, SPC25, CENPE, LYPD3, SRPX2, CDC6, DEPDC1-AS1, PBK, RALGPS2, KIF18B, SKA3, LOC100129029, CDCA3, CDKN3, TIMP1, CDC25C, KIAA1024, FUT9, UBE2C, PRPF31, CCNB2, MKI67, MFAP5
TCGA MMPs-gene cluster (n=185)	TMPRSS11E2, ABCA12, ABCA4, ABCC3, ACY3, ADAM12, ADAM28, ADAMDEC1, ADAMTS14, ADAMTS16, AIM2, AK3L1, ALPK2, ANKRD22, APCDD1L, ARNTL2, ATP10B, B3GNT3, B3GNT6, BARX2, BCL2L15, BMP8A, C11orf86, C15orf48, C1orf170, C1QTNF6, C20orf151, C2CD4A, C4orf7, C5orf46, C6orf222, C8orf73, C9orf84, CA9, CD19, CP, CBLC, CDH3, CEACAM5, CEACAM7, CILP2, COL10A1, COL11A1, COL17A1, COL1A1, COL22A1, COL3A1, COL7A1, COMP, CPNE4, CPXM1, CR2, CST1, CST2, CST4, CTHRC1, CXCL13, CXCL14, CYP24A1, CYP27C1, DERL3, DIO2, DNAJC22, DPEP1, EGLN3, EPHA10, EPHX4, EPN3, EPYC, FAM155B, FAM83A, FAP, FBXO32, FCRL5, FERMT1, FEZF1, FHL2, FLJ40330, FOXP3, GALNT14, GJB2, GJB6, GLB1L3, GOLM1, GPR115, GPR87, GREB1L, GREM1, GRHL3, HMGA2, HNF4G,

	HS6ST2, HTR3A, IBSP, IGFL2, IL11, IL1F5, IL1F7, IL1RL2, IL20RB, IL22RA2, IL31RA, ITGA11, ITPKA, JSRP1, KCNN4, KCNQ3, KDELR3, KIF26B, KPNA7, KRT80, LGR4, LOC100131726, LOC285629, LOC84740, LOC96610, LYPD1, METTL7B, MFI2, MMP1, MMP10, MMP11, MMP12, MMP13, MMP3, MMP9, MUC16, MYEOV, MYO3B, NETO1, NGEF, NMU, NRK, OCIAD2, ONECUT1, ONECUT2, OR51E1, OTX1, OVOL1, P4HA3, PADI1, PGLYRP4, PHLDA2, PITX1, PLEK2, PLEKHN1, PODNL1, PPAP2C, PPAPDC1A, PROM2, PRSS3, PTGES, PVRL4, PVT1, RGS17, RHBDL2, RHOV, S100A2, SHOX2, SLC22A18AS, SLC2A1, SLC2A5, SLCO1B3, SPRR1B, SPTBN2, STEAP1, STYK1, SULF1, SYT12, TCN1, TFAP2A, THBS2, TMEM156, TMEM184A, TMEM63C, TRIM15, TRIM31, TRPM8, VPREB3, VSIG1, XAGE1D, XDH, XKRX, ZIC2, ZPLD1
MMPs-gene signature (n=36)	COL11A1, MMP13, MMP12, GJB2, CYP24A1, GREM1, MMP11, MMP1, IGFL2, MMP3, MUC16, MMP9, CTHRC1, COL17A1, KIF26B, GPR87, ZIC2, IBSP, KCNN4, THBS2, C15orf48, FAM83A, S100A2, SULF1, EPYC, PPAPDC1A, ALPK2, PITX1, SLC2A1, ADAM12, STEAP1, EGLN3, RHOV, FHL2, LYPD1, TCN1

The MMP gene clusters were determined from GSE31210 and TCGA lung adenocarcinoma cohort, respectively. The MMP common gene signature (n=36) was identified by the overlapping genes between GSE31210 and TCGA cohort MMP gene cluster. *Definition of abbreviations*: MMP = matrix metalloproteases.

.

Table S3. Major Pathways in 150-gene cluster from GSE31210 cohort by IPA.

Ingenuity Canonical Pathways	-log(*p*-value)	Molecules
Matrix Metalloproteases	11.44	MMP3,ADAM12,TIMP1,THBS2,MMP13, MMP11, MMP12,MMP9,MMP1
HIF1α Signaling	5.56	MMP3,SLC2A1,MMP13,EGLN3,MMP11, MMP12,MMP9,MMP1
Bladder Cancer Signaling	4.28	MMP3,MMP13,MMP11,MMP12,MMP9,M MP1
Leukocyte Extravasation Signaling	4.01	MMP3,TIMP1,CLDN1,MMP13,MMP11,M MP12, MMP9,MMP1
Granulocyte Adhesion and Diapedesis	3.84	MMP3,CLDN1,MMP13,MMP11,MMP12,M MP9, MMP1
Oncostatin M Signaling	3.67	MMP3,MMP13,PLAU,MMP1
Glioma Invasiveness Signaling	3.45	RHOV,TIMP1,HMMR,PLAU,MMP9
Colonrectal Cancer Metastasis	3.36	MMP3,MMP13,MMP11,MMP12,MMP9,M MP1, BIRC5,RHOV

The MMP gene cluster, which contained 150 MMP related gene were determined from GSE31210 stage I-II lung adenocarcinoma cohort for Ingenuity canonical pathways (IPA) analysis. Ingenuity canonical pathways were combined according to the major function category and gene overlap. The enrichment *p*-values were obtained by Fisher Exact Test. Pathways were ranked within the category by –log(*p*-value) for the pathway. –log(*p*-value) >1.3 with significance at *p*=0.05. *Definition of abbreviations*: ADC = adenocarcinoma; MMP = matrix metalloproteases.

Table S4. Clinical Characteristics of TCGA Lung Adenocarcinoma Cohort .

**Characteristics**	***N* (%)**
Subjects, *n*	517
Sex, M/F, *n* (%)	240/277 (46/54)
Age, yr, mean ± SD	65 ± 13
pTNM stages*	
I	277 (54)
II	122 (24)
III	84 (16)
IV	26 (5)
NA	8 (1)
Smoking status	
Non-smoker	76 (15)
Ex-smoker	308 (60)
Current-smoker	119 (23)
NA	14 (2)
Mutation status	
EGFR_MUT	66 (13)
KRAS_MUT	154 (30)
ALK _Fusion	5 (1)
KRAS/EGFR/AKL_WT	285 (55)
NA	7 (1)
Treatment	
Surgery	386 (75)
Radiotherapy	43 (8)
Chemotherapy	155 (30)
Targeted therapy	18 (3)

Clinical–pathologic characteristics of TCGA lung adenocarcinoma cohort (n=517) were evaluated in this study. *According to the 6th and 7th edition of the TNM classification for lung cancer. *Definition of abbreviations:* ALK: Anaplastic lymphoma kinase; EGFR= epidermal growth factor receptor; MUT = mutation; NA = not applicable; pTNM stages = pathologic TNM stages of malignant tumors; TCGA = the cancer genome atlas; WT = wild type.

Table S5. Major Pathways in 185-gene cluster from TCGA lung adenocarcinoma cohort by IPA.

Ingenuity Canonical Pathways	-log(*p*-value)	Molecules
Matrix Metalloproteases	10.46	MMP3,ADAM12,THBS2,MMP10,MMP13, MMP11,MMP12,MMP9,MMP1
Granulocyte Adhesion and Diapedesis	7.55	MMP3,CXCL13,IL1RL2,IL36RN,CXCL14, MMP10,MMP13,MMP11,IL37,MMP12, MMP9,MMP1
HIF1α Signaling	6.70	SLC2A5,MMP3,SLC2A1,EGLN3,MMP10, MMP13,MMP11,MMP12,MMP9,MMP1
Agranulocyte Adhesion and Diapedesis	6.30	MMP3,CXCL13,IL36RN,CXCL14,MMP10, MMP13,MMP11,IL37,MMP12,MMP9,MM P1
Hepatic Fibrosis / Hepatic Stellate Cell Activation	6.16	COL1A1,IL1RL2,COL10A1,COL22A1,MM P13, COL11A1,COL17A1,MMP9,MMP1,COL7 A1, COL3A1
Atherosclerosis Signaling	5.76	COL1A1,MMP3,IL36RN,COL10A1,MMP1 3,IL37,MMP9,MMP1,COL3A1
Bladder Cancer Signaling	4.63	MMP3,MMP13,MMP10,MMP11,MMP12, MMP9,MMP1
Axonal Guidance Signaling	4.57	NGEF,ADAMTS14,MMP3,BMP8A,MMP1 0, MMP13,EPHA10,ADAMDEC1,ADAM12, ADAM28,MMP11,MMP12,ADAMTS16,M MP9, MMP1
Osteoarthritis Pathway	4.02	MMP3,IL1RL2,COL10A1,MMP10,MMP13, GREM1,MMP12,MMP9,MMP1

The MMP gene signature, which contained 185 MMP related genes were determined from TCGA lung adenocarcinoma cohort for Ingenuity canonical pathways (IPA) analysis. Ingenuity canonical pathways were combined according to the major function category and gene overlap. The enrichment *p*-values were obtained by Fisher Exact Test. Pathways were ranked within the category by –log(*p*-value) for the pathway. –log(*p*-value) >1.3 with significance at p=0.05.

Table S6. Major Pathways in 36-gene MMP signature by IPA.

Ingenuity Canonical Pathways	-log(*p*-value)	Molecules
Matrix Metalloproteases	14.66	ADAM12, MMP1, MMP3, MMP9, MMP11, MMP12, MMP13, THBS2
HIF1α Signaling	10.27	EGL3, MMP1, MMP3, MMP9, MMP11, MMP12, MMP13, SLC2A1
Granulocyte Adhesion and Diapedesis	6.35	MMP1, MMP3, MMP9, MMP11, MMP12, MMP13
Osteoarthritis Pathway	5.79	GREM1, MMP1, MMP3, MMP9, MMP12, MMP13
Hepatic Fibrosis/Hepatic Stellate Cell Activation	4.76	COL11A1, COL17A1, MMP1, MMP9, MMP13
Axonal Guidance Signaling	4.70	ADAM12, MMP1, MMP3, MMP9, MMP11, MMP12, MMP13
Oncostatin M Signaling	4.21	MMP1, MMP3, MMP13
Atherosclerosis Signaling	4.14	MMP1, MMP3, MMP9, MMP13

The 36-gene MMP signature, which contained overlapping genes of MMP cluster genes between GSE31210 and TCGA lung adenocarcinoma cohort was determined for Ingenuity canonical pathways (IPA) analysis. Ingenuity canonical pathways were combined according to the major function category and gene overlap. The enrichment *p*-values were obtained by Fisher Exact Test. Pathways were ranked within the category by –log(*p*-value) for the pathway. –log(*p*-value) >1.3 with significance at p=0.05.

Table S7. Univariate and Multivariate Analysis of Progression-free and Overall Survival in 162 stage I lung adenocarcinoma.

**Variable**	**Progression-free Survival**				**Overall Survival**			
	Univariate Analysis		Multivariate Analysis		Univariate Analysis		Multivariate Analysis	
	hazard ratio (95% CI)	*P value*	*hazard ratio (95% CI)*	*P value*	*hazard ratio (95% CI)*	*P value*	*hazard ratio (95% CI)*	*P value*
Sex, M	0.99 (0.50–1.96)	0.98	0.78 (0.33–1.83)	0.56	1.14 (0.43–3.07)	0.88	0.84 (0.23–3.03)	0.79
Age, per 1 yr*	1.01 (0.96–1.06)	0.67	0.99 (0.96–1.06)	0.68	0.99 (0.93–1.06)	0.73	0.97 (0.91–1.04)	0.38
Stage IB vs IA	3.02 (1.53–5.95)	0.001	2.18 (1.06–4.5)	**0.03**	2.16 (0.81–5.78)	0.12	1.25 (0.43–3.61)	0.68
Smoking status								
Ever-smoker vs. Never-smoker	1.06 (0.54–2.08)	0.87	0.93 (0.39–2.23)	0.86	1.46 (0.55–3.89)	0.45	1.11 (0.30–4.02)	0.88
Mutation status								
EGFR_MUT vs. KRAS/EGFR/AKL_WT	0.44 (0.22–0.92)	**0.029**	0.61 (0.29–1.28)	0.19	0.35 (0.13–0.96)	**0.042**	0.48 (0.17-1.40)	0.18
KRAS_MUT vs. KRAS/EGFR/AKL_WT	0.84 (0.28–2.55)	0.76	0.60 (0.19–1.91)	0.39	0.35 (0.04–2.80)	0.32	0.29 (0.04–2.42)	0.26
ALK _Fusion vs. KRAS/EGFR/AKL_WT	$9.40e^{-7}$ (0–Inf)	0.99	$1.27e^{-10}$ (0–Inf)	0.99	$6.37e^{-9}$ (0–Inf)	0.99	$1.44e^{-10}$ (0-Inf)	0.99
MMP gene signature								
High vs Low	3.89 (1.97–7.67)	**0.04**	3.14 (1.49–6.60)	**0.003**	6.43 (2.23–18.53)	**0.0006**	6.00 (1.92–18.72)	**0.002**

All variables were evaluated among the 162 patients with lung adenocarcinoma (6 patients excluded from 168 patients from GSE31210 cohort due to exclude for prognosis analysis due to incomplete resection or adjuvant therapy).

Factors associated with PFS and OS in univariate and multivariate Cox regression model (n=162 patients). The HR (95%CI) and *P* value are shown for each. *P* value <0.05 are set in bold for emphasis.

*Additional risk with each additional year of age.

Definition of abbreviations: ALK = Anaplastic lymphoma kinase; EGFR=epidermal growth factor receptor; Inf = Infinite; KRAS=Kirsten rat sarcoma 2 viral oncogene homolog; MMP=metalloproteinase; MUT=mutation; WT= wild type.

Table S8. Clinical Characteristics of GSE30219 Stage I Lung Adenocarcinoma Cohort .

Characteristics	*N* (%)
Subjects, *n*	70
Sex, M/F, *n* (%)	54/16 (77/23)
Age, yr, mean ± SD	61 ± 9
pTNM stages	
T1N0M0	70 (100)
Tobacco	7
Never-smoker	75 (7)
Former-smoker	34 (49)
Active-smoker	30 (43)
NA	1 (1)
TP53 mutation	
WT	42 (60)
MUT	25 (36)
NA	3 (4)
Adjuvant chemotherapy	
No	68 (97)
Yes	0 (0)
NA	2 (3)
Adjuvant radiotherapy	
No	69 (99)
Yes	0 (0)
NA	1 (1)

Clinical–pathologic characteristics of GSE30219 lung adenocarcinoma cohort (n=70) were evaluated in this study. *According to the 7th edition of the TNM classification for lung cancer. Definition of abbreviations: MUT = mutation; NA = not applicable; pTNM stages = pathologic TNM stages of malignant tumors; WT = wild type.
